# Epigenetic Regulation of Myogenic Gene Expression by Heterochromatin Protein 1 Alpha

**DOI:** 10.1371/journal.pone.0058319

**Published:** 2013-03-11

**Authors:** Patima Sdek, Kyohei Oyama, Ekaterini Angelis, Shing S. Chan, Katja Schenke-Layland, W. Robb MacLellan

**Affiliations:** Departments of Medicine/Cardiology, Center for Cardiovascular Biology, Institute for Stem Cell Research, University of Washington School of Medicine, Seattle, Washington, United States of America; St Jude Children’s Research Hospital, United States of America

## Abstract

Heterochromatin protein 1 (HP1) is an essential heterochromatin-associated protein typically involved in the epigenetic regulation of gene silencing. However, recent reports have demonstrated that HP1 can also activate gene expression in certain contexts including differentiation. To explore the role of each of the three mammalian HP1 family members (α, β and γ) in skeletal muscle, their expression was individually disrupted in differentiating skeletal myocytes. Among the three isoforms of HP1, HP1α was specifically required for myogenic gene expression in myoblasts only. Knockdown of HP1α led to a defect in transcription of skeletal muscle-specific genes including Lbx1, MyoD and myogenin. HP1α binds to the genomic region of myogenic genes and depletion of HP1α results in a paradoxical increase in histone H3 lysine 9 trimethylation (H3K9me3) at these sites. JHDM3A, a H3K9 demethylase also binds to myogenic gene’s genomic regions in myoblasts in a HP1α-dependent manner. JHDM3A interacts with HP1α and knockdown of JHDM3A in myoblasts recapitulates the decreased myogenic gene transcription seen with HP1α depletion. These results propose a novel mechanism for HP1α-dependent gene activation by interacting with the demethylase JHDM3A and that HP1α is required for maintenance of myogenic gene expression in myoblasts.

## Introduction

Skeletal muscle differentiation is a multistep process which begins with the commitment of multi-potent mesodermal precursor cells to the muscle fate and culminates with the upregulation of skeletal muscle-specific genes, permanent cell cycle withdrawal and silencing of genes required for cell cycle progression in myotubes [1], [2], [3]. The muscle regulatory factors that drive myogenic differentiation have been well studied; however, the mechanisms that control their expression are poorly understood. It has been suggested that epigenetic regulation and post-translation modifications of histones in particular play a critical role in skeletal muscle differentiation [4], [5].

HP1s are a family of essential heterochromatin-associated proteins that recognize methylated histones and classically play a role in the organization of heterochromatin formation and gene silencing in many organisms [6]. Mammalian cells contain three HP1 isoforms: HP1α, HP1β and HP1γ, which differ in localization and interaction partners and thus likely have distinct cellular functions [7], [8]. The mechanism of HP1-dependent gene silencing has been extensively studied and occurs via the formation of heterochromatin at hetero- and euchromatic promoters. Conversely, recent reports indicate that HP1 can also activate euchromatic and heterochromatic gene expression in certain situations [9], [10], [11]. The mechanism of HP1-dependent gene expression activation remains largely unclear but in contrast to its association with promoters when silencing genes, HP1 can associate anywhere within the genomic sequences when activating gene expression.

The role of HP1 family members during differentiation including skeletal muscle has had limited investigation [12], [13], [14], [15], [16], [17]. Recent reports, based primarily on heterologous systems, suggest that HP1 proteins might negatively regulate skeletal muscle differentiation by inhibiting skeletal muscle-specific factors, MEF2 and MyoD in myoblasts [12], [16]. However, when endogenous HP1 expression was depleted, instead of activating MyoD-dependent genes, skeletal muscle differentiation was inhibited [16]. The basis for this paradox was not resolved; however, it was postulated that it might be an indirect effect related to a failure to downregulate proliferation-associated genes although this was not shown.

In order to explore the mechanism(s) underlying the dual functions of HP1 in skeletal muscle differentiation, we disrupted the expression of each HP1 family member in differentiating skeletal myocytes. Among the three isoforms of HP1, HP1α was specifically required for myogenic differentiation and blocking its expression led to a defect in the transcription of skeletal muscle-specific genes including Lbx1, MyoD and myogenin. This defect was not secondary to aberrant expression of cell cycle-associated genes. Instead, HP1α appears to regulate H3K9me3 demethylation of target myogenic genes by interacting with the histone demethylase JHDM3A thus facilitating gene expression. Therefore, our results suggest a bifunctional role for HP1α in skeletal myoblasts designed to maintain their committed but undifferentiated state. This study suggests a novel mechanism for HP1α-dependent myogenic gene expression.

## Results

### Expression and Nuclear Distribution of HP1 Proteins during Skeletal Muscle Differentiation

**Figure 1:**
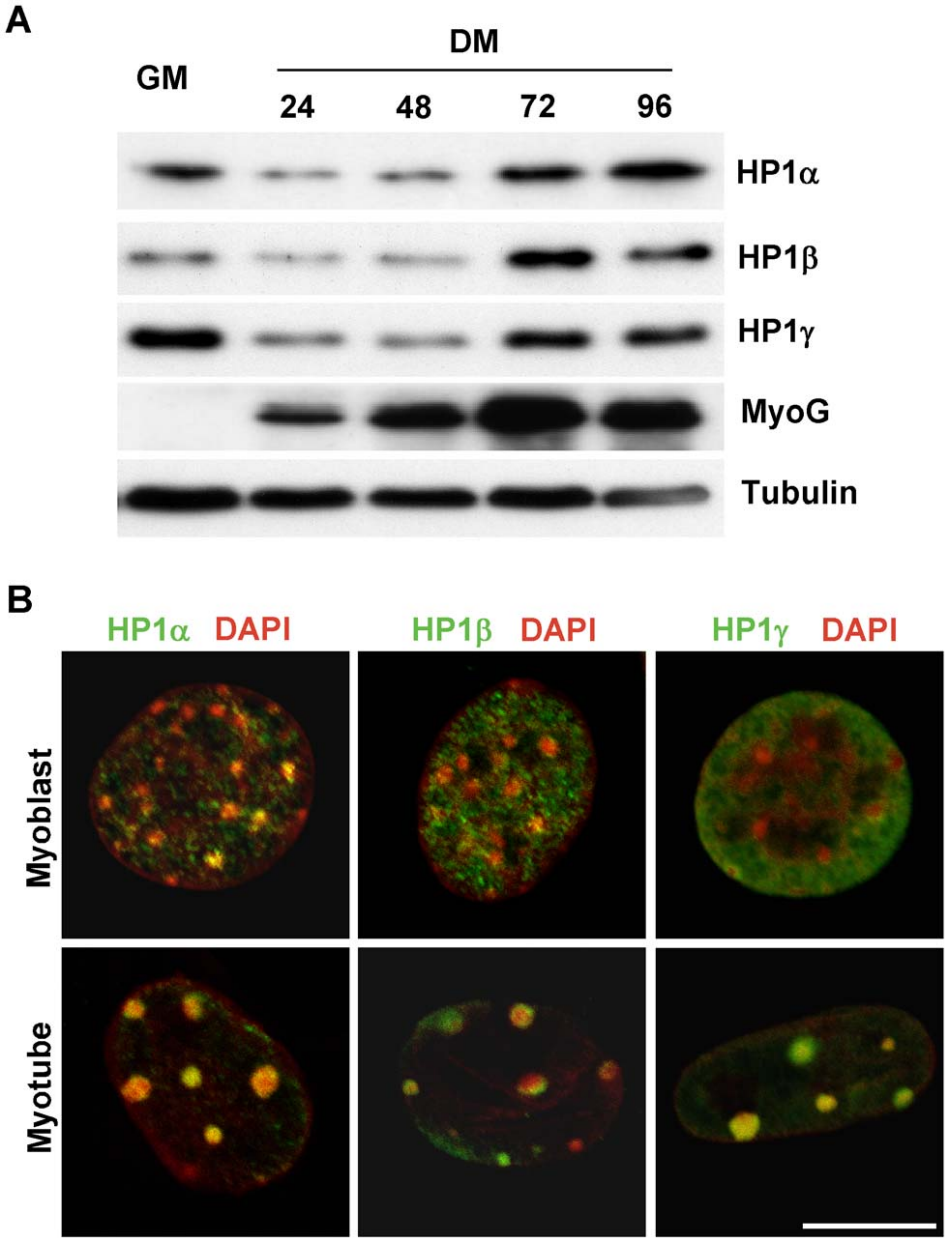
Expression and nuclear distribution of HP1 proteins during skeletal muscle differentiation. *A.* C2C12 cells were cultured in GM (growth medium) or DM (differentiation medium) and collected at indicated time points. Total protein was extracted and subjected to Western blotting. *B*. Confocal fluorescence microscopy was performed on C2C12 myoblasts and myotubes after immunostaining for the indicated HP1 protein (green) and DAPI (converted to red). Scale bar equals 10 µm.

To explore the role of HP1s in regulating skeletal muscle differentiation, we examined HP1 protein expression at serial time points during differentiation of C2C12 cells, a clonal skeletal myoblast cell line. All three HP1 family members were expressed in skeletal muscle although their developmental pattern of expression differed. HP1α and HP1γ displayed a similar biphasic expression pattern; namely, downregulation upon initiation of differentiation with subsequent upregulation in myotubes. In contrast, HP1β protein levels were low in myoblasts but were upregulated in myotubes (Fig. 1A). As expected, myotubes demonstrated increased myogenin expression. To determine the nuclear distribution of HP1 proteins, we examined myoblasts and myotubes with antibodies to HP1 proteins and imaged the nuclear DNA with DAPI (Fig. 1B, Fig. S1). It has been suggested that pericentric heterochromatin aggregates develop during myogenic differentiation, which can be identified by concentrated DAPI staining [18], [19]. Heterochromatin aggregates increased dramatically in myotubes although limited, small dense chromatin areas are also apparent in myoblasts (Fig. 1B). In myoblasts, HP1α and HP1β were distributed throughout both lighter stained euchromatic regions and densely stained heterochromatic areas while HP1γ was exclusively localized to euchromatin. However, all HP1 family members colocalized with heterochromatin in differentiated myotubes. These differing temporal and subnuclear expression patterns suggest that the function of HP1 isoforms may differ not only between family members but also on the developmental time point.

### Knockdown of HP1α, but not HP1β and HP1γ, Blocks Skeletal Muscle Differentiation

**Figure 2:**
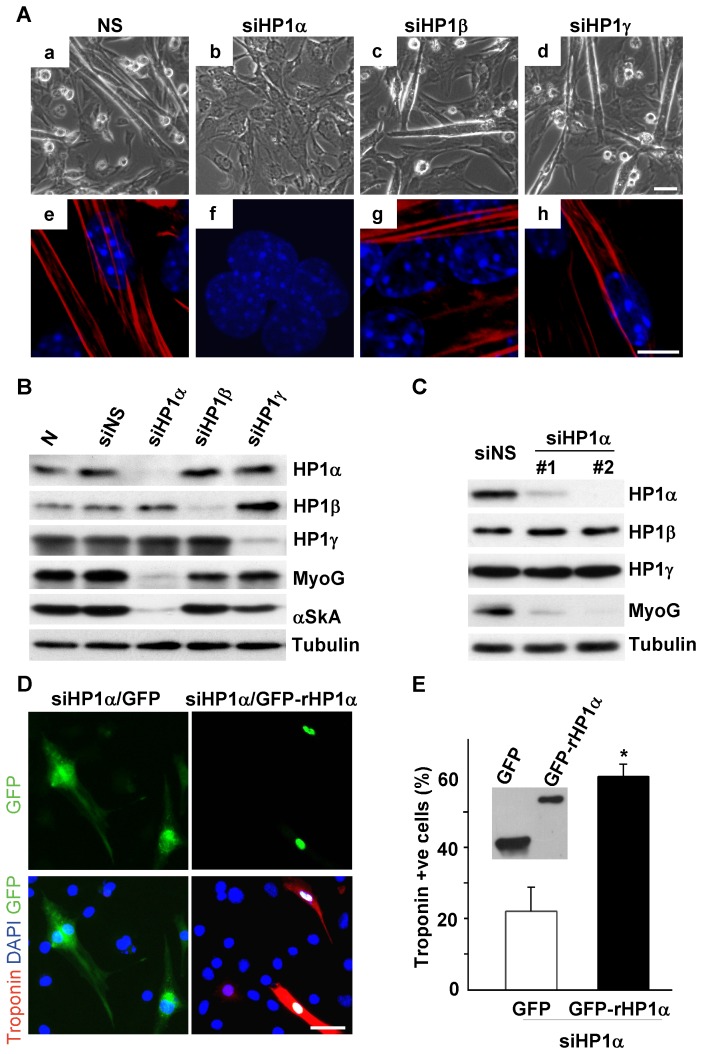
HP1α is required for skeletal muscle differentiation. C2C12 cells were transfected with the indicated siRNA in GM and 24 hours after transfection, DM was added and cultured for 72 hours. *A.* Phase contrast images were obtained (A a–d). Confocal fluorescence microscopy was performed after immunostaining for skeletal sarcomeric myosin heavy chain (red) and DAPI (blue) (A e–h). Scale bar equals 50 µm (A a–d) and 6 µm (A e–h). *B,* Total protein lysates were subjected to Western blotting. *C.* C2C12 cells were transfected with NSsiRNA or HP1αsiRNA (#1: ID# 60593; #2: ID# 60497) and cultured in DM for 72 hours. Total protein lysates were subjected to Western blotting. *D.* Reintroduction of siRNA resistant version of HP1α rescued myogenic differentiation in HP1αsiRNA transfected cells. C2C12 myoblasts were co-transfected with the indicated siRNA and pLPC-EHGF or pLPC-EHGF-rHP1α plasmids. 24 hours after transfection, DM was added and cultured for another 72 hours. Cells were stained with anti-GFP (green) and anti-Troponin C (red), nuclei were counterstained with DAPI (blue). Scale bar equals 50 µm. *E*. The total number of GFP positive cells and the cells positive for both GFP and Troponin C were counted and percentage of Troponin C positive cells in GFP positive cells were calculated. *P<0.01 for siHP1α/GFP versus siHP1α/GFP-rHP1α cells. Expression of GFP and GFP fusion HP1 were detected by Western blotting using anti-GFP antibody.

To determine the effects of depleting HP1 family members on skeletal muscle differentiation, we inhibited HP1 expression with oligonucleotide siRNAs to each of the individual HP1 family members (HP1α, -β and -γ). Myoblasts were transfected with each specific HP1 siRNA, after which myotube formation or myogenic gene expression was examined after 72 hours of culturing in differentiation media (DM). Phase contrast images of C2C12 cells show that cells transfected with nonspecific siRNA (NSsiRNA) as well as cells transfected with HP1β and HP1γ siRNA formed myotubes normally (Fig. 2A a, c, d); however, myotube formation was absent in cells deficient for HP1α (Fig. 2A b). Expression of skeletal sarcomeric α-actinin, a marker of differentiation, and heterochromatin aggregation was dramatically impaired in HP1α deficient cells compared to NSsiRNA transfected cells (Fig. 2A–f vs. e). HP1α, -β and -γ were efficiently and specifically knocked down, myogenin and α-skeletal actin expression was blocked exclusively in HP1α depleted myocytes (Fig. 2B). To ensure that the effect of depleting HP1α on myoblast differentiation was specific, we confirmed these results using a second independent siRNA having different target sequences within HP1α. Both siRNAs efficiently reduced the level of HP1α, leading to marked reductions in myogenin levels and impaired differentiation (Fig. 2C).

To further confirm the specificity of the effect of depleting HP1α on skeletal muscle differentiation, we determined if the defect in differentiation could be rescued by re-introducing HP1α. We generated a siRNA-resistant GFP-HP1α fusion protein (GFP-rHP1α) by mutating four nucleotides in the HP1αsiRNA target sequence that conserved the amino acid sequence. Relative expression of GFP and GFP-rHP1α fusion protein are demonstrated by immunostaining and Western blotting (Fig. 2 D & E). siRNA oligonucleotides and GFP expressing plasmids (GFP or GFP-rHP1α) were co-transfected in C2C12 myoblasts and the cells were induced to differentiate. Differentiated cells were identified by Troponin C immunostaining (Fig. 2D). The percentage of Troponin C positive cells in GFP-rHP1α expressing cells was significantly higher as compared to GFP-only cells (60% *vs.* 22%, *P* = 0.007; Fig. 2E). These data demonstrate that HP1α is specifically required for skeletal muscle differentiation.

### HP1α-deficient Myocytes Demonstrate a Defect at the Committed Myoblast Stage with Reduced Myogenic Gene Expression

**Figure 3:**
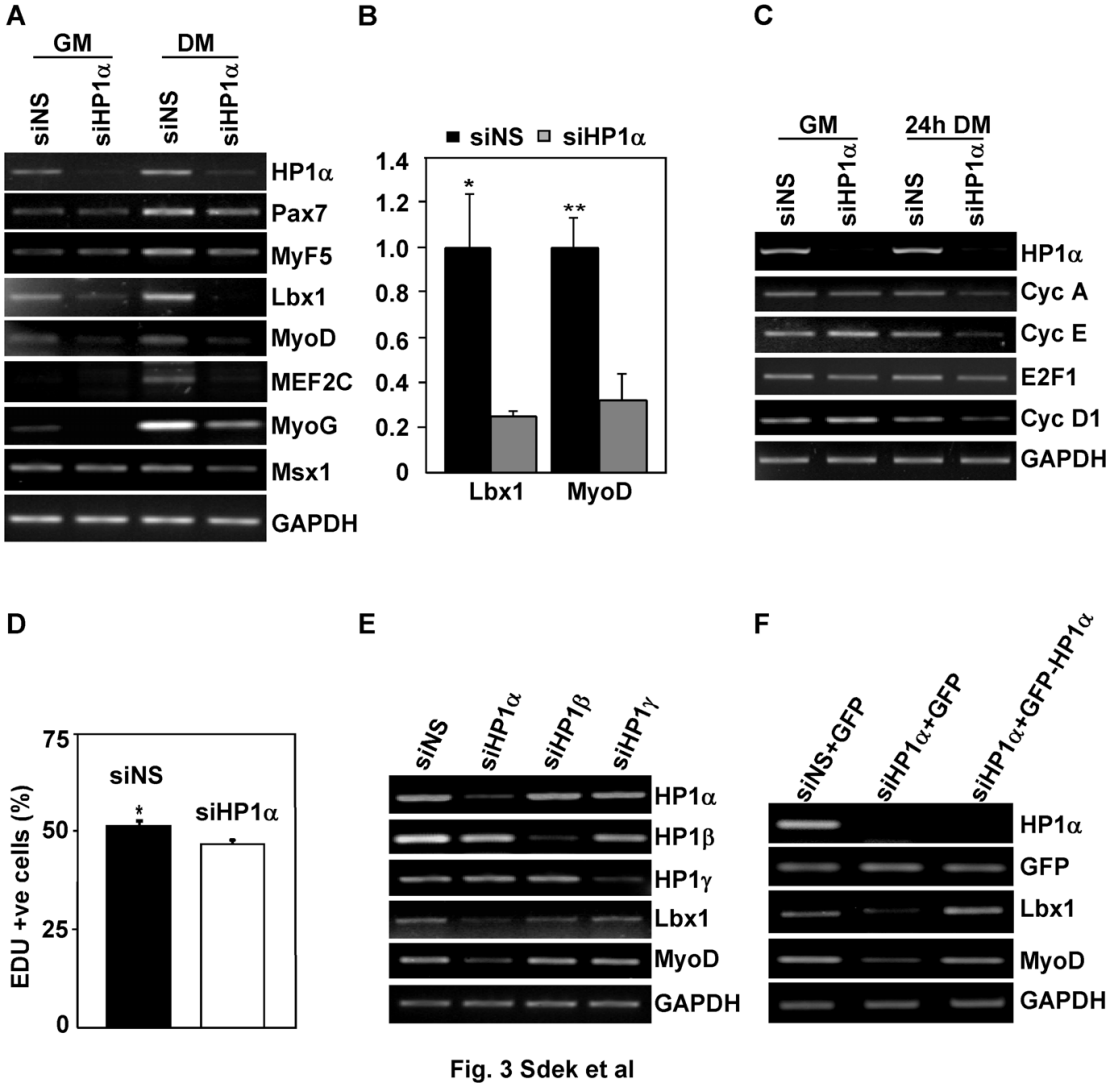
HP1α-deficient myocytes demonstrate a defect at the committed myoblast stage with reduced Lbx and MyoD expression. *A, B, C.* C2C12 myoblasts were transfected with indicated siRNA. 24 hours after transfection, transfection medium were changed to GM or DM, cells were incubated for an additional 24 hours. Total RNA was isolated and semiquantitative PCR analysis of gene expression was performed (A, C). mRNA levels of Lbx1 and MyoD (B) in cells cultured in GM quantified by real-time PCR normalized to GAPDH. *P<0.05 for Lbx1 levels siNS vs. siHP1α; **P<0.05 for MyoD levels siNS vs. siHP1α. *D*. EDU assay were performed 24 hours after indicated siRNA transfection in C2C12 myoblasts. Percentage of EDU positive cells was calculated. *P<0.05 for sins versus siHP1α. *E.* C2C12 cells were transfected with indicated siRNA. Total RNA was isolated 36 hours after transfection. Semiquantitative PCR analysis of gene expression was performed. *F.* C2C12 myoblasts were co-transfected with indicated siRNA and GFP-expressing plasmid. Total RNA was extracted from GFP-positive cells isolated by fluorescence-activated cell sorting (FACS). Semiquantitative PCR analysis of gene expression was performed.

To determine the basis for the defect in myogenic differentiation created by HP1α deficiency, transcript levels of a panel of genes involved in myogenic differentiation were examined after HP1αsiRNA transfection in C2C12 cells (Fig. 3A, B, C). Transcript levels of Pax7 and Myf-5 were minimally affected after HP1α knockdown while Lbx1, MyoD, MEF2C and myogenin levels were markedly decreased (Fig. 3A and B). Expression of Msx1, which is known to inhibit skeletal muscle differentiation through repression of myogenic genes including MyoD [20] was not altered significantly in HP1α–deficient myoblasts. It has been proposed that the myogenic defect caused by depletion of HP1α might related to a failure to downregulate proliferation-associated genes [16], which is necessary for cell cycle exit during terminal differentiation. In contrast to this hypothesis, we detected a slight downregulation, not upregulation of E2F-dependent target genes after depleting HP1α (Fig. 3C). To further clarify the effect of depleting HP1α on cell proliferation, we assayed DNA synthesis using a novel thymidine analogue, 5-Ethynyl-2′-deoxyuridine (EDU). C2C12 myoblasts transfected with NSsiRNA showed 51% of the cells incorporated EDU while depletion of HP1α decreased the number of EDU-labeled cells to 46% (Fig. 3D & Fig. S2). This finding is consistent with expression levels of E2F-dependent genes in HP1α knockdown cells and confirms that the defect in myogenic differentiation observed in HP1 deficient cells is not a result of persistent expression of proliferation-associated genes. Consistent with our earlier results, C2C12 cells deficient for HP1β and -γ expressed normal levels of myogenic genes (Fig. 3E). This defect in myogenic gene expression could be rescued by restoring HP1α expression. GFP or the siRNA-resistant GFP-rHP1α fusion protein was overexpressed in HP1αsiRNA transfected cells and GFP-positive cells were isolated by fluorescence-activated cell sorting (Fig. S3). Lbx1 and MyoD mRNA levels were upregulated in GFP-rHP1α expressing cells compared to GFP-only expressing cells (Fig. 3F). These data suggest the effect of HP1α on skeletal muscle differentiation might be secondary to the direct regulation of myogenic gene transcription.

### HP1α is Dispensable for Myogenic Gene Expression and Maintenance of Terminal Differentiation in Myotubes

**Figure 4:**
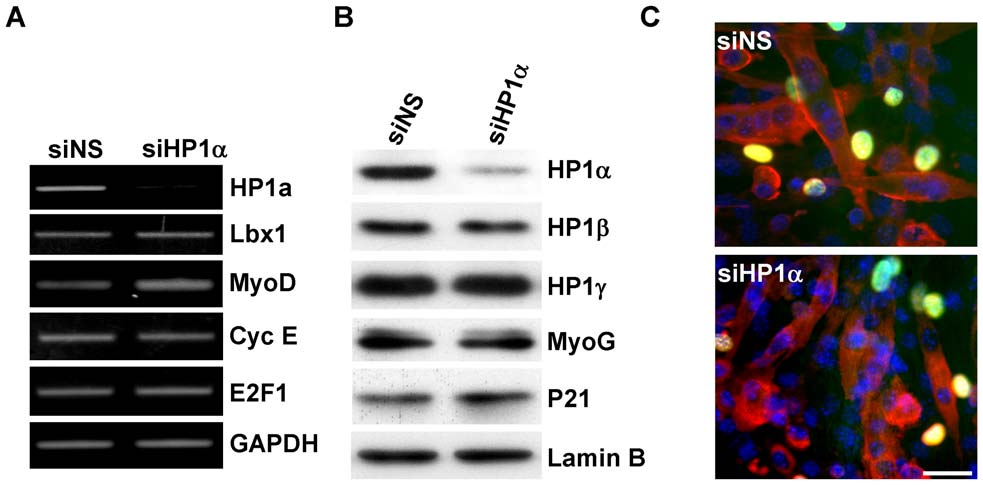
HP1α is dispensable for maintenance of terminal differentiation. C2C12 cells were cultured in DM for 72 hours and then transfected with indicated siRNA. *A*, 48 hours after transfection, total RNA was isolated and semiquantitative PCR analysis of gene expression was performed. *B.* 24 hours after siRNA transfection, DMEM contain 20% FBS were added and cells were cultured for another 24 hours. Total protein were extracted and probed for indicated antibodies. *C.* 24 hours after siRNA transfection, cells were stimulated with DMEM contain 20% FBS in the presence of BrdU for 24 hours. Cell cycle reentry was determined by immunostaining for BrdU incorporation (Blue: DAPI, Red: sarcomeric myosin heavy chain; Green; BrdU). Scale bar equals 50 µm.

Although our results demonstrate a critical role for HP1α in myoblast gene transcription, its role in myotubes was uncertain. The fact that HP1α is expressed and colocalizes with heterochromatin in myotubes (Fig. 1B & Fig. S1) suggested it might have a distinct role at this developmental time point. Thus, to investigate the effect of HP1α on myogenic and E2F-dependent gene expression in myotubes, we inhibited HP1α expression specifically in myotubes, bypassing the defect elicited in myoblasts. The transfection efficiency of oligonucleotide siRNA in myotubes was greater than 95% when tested by using CY3-labled siRNA (data not shown). HP1αsiRNA, even when delivered after myocyte differentiation, efficiently reduced HP1α RNA and protein levels (Fig. 4A & B); however, Lbx1 and MyoD expression were not downregulated as was seen in myoblasts (Fig. 4A) nor were the levels of late markers of myogenic differentiation such as myogenin or p21 affected (Fig. 4B). Likewise, there were no significant changes in expression of cell cycle associated factors Cyclin E and E2F1 (Fig. 4A). To determine if HP1α was required for the maintenance of the terminally differentiated state, siRNA transfected myotubes were stimulated with 20% FBS for 24 hours in the presence of BrdU to label myotubes entering S phase. No increase in BrdU-labeled myotubes was seen after serum stimulation suggesting that HP1α-depleted myotubes were not capable of reentering the cell cycle (Fig. 4C).

### Histone Lysine 9 Trimethylation (H3K9me3) in Myogenic Gene Coding Regions is Increased after Depleting HP1α

**Figure 5:**
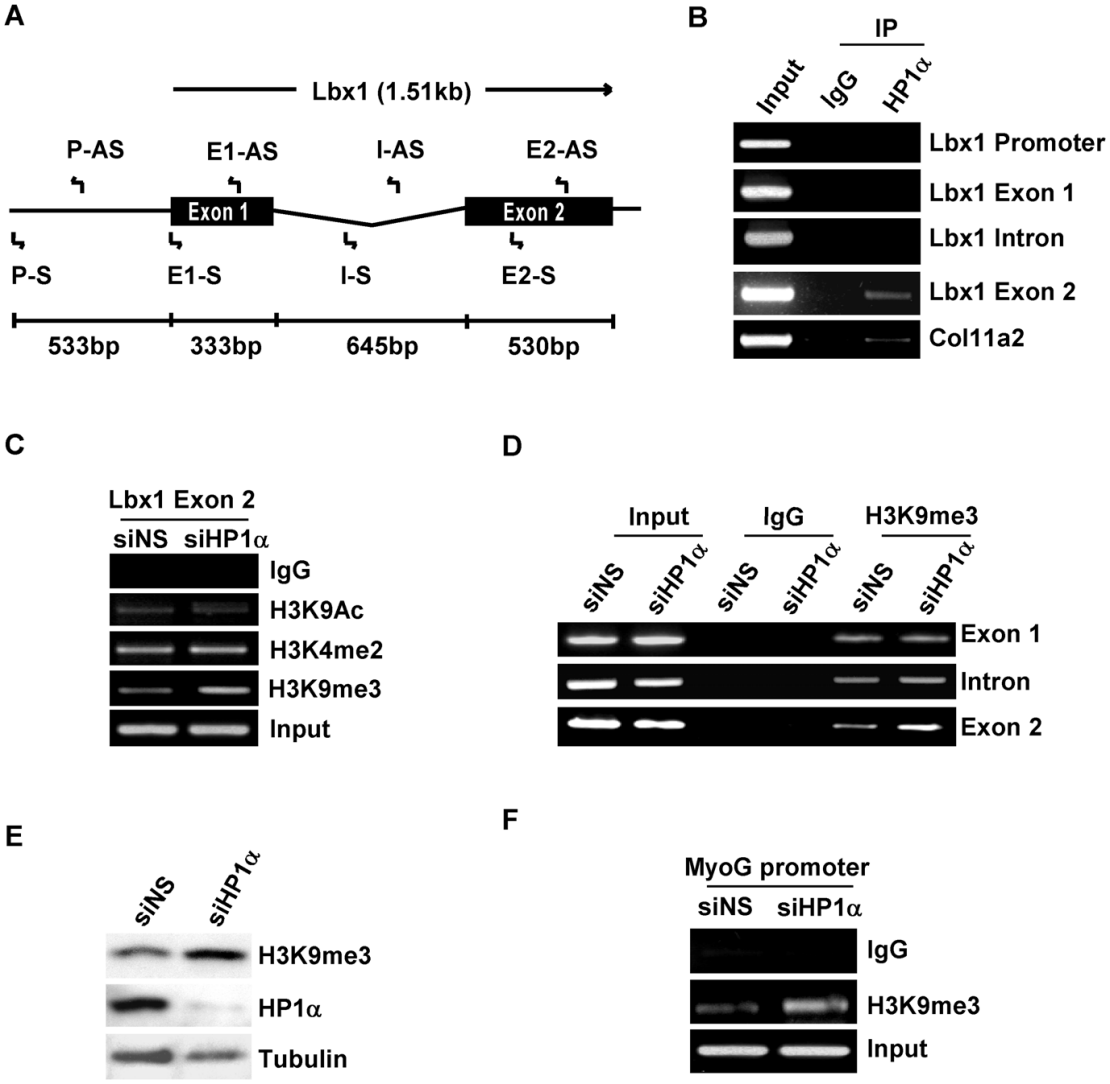
H3K9me3 levels on myogenic genes increased in C2C12 myoblasts after depleting HP1α. *A.* Schematic diagram of the genomic structure of the mouse Lbx1 gene and locations of primers used for subsequent ChIP experiments. *B.* Protein-DNA complexes from cross-linked chromatin extracted from C2C12 myoblasts cultured in GM were immunoprecipitated with HP1α or mouse IgG. Bound DNA was amplified using the indicated PCR primers. *C, D,* C2C12 myoblasts were transfected with indicated siRNA, 48 hours after transfection, cross-linked chromatin was extracted and immunoprecipitated with indicted antibodies. *Lbx1* exon 2 (C) or *Lbx1* genomic sequences including exon 1, intron and exon 2 (D) were amplified. E. C2C12 myoblasts were transfected with the indicated siRNA, 48 hours after transfection total cell lysates were subjected to Western blotting with the indicated antibodies. *F.* C2C12 myoblasts were transfected with indicated siRNA, 48 hours after transfection cross-linked chromatin was extracted and immunoprecipitated with anti-H3K9me3 antibody. Precipitated DNA was used for PCR with primers spanning the MEF2-binding site on the myogenin gene promoter.

It has been suggested that HP1 can associate anywhere within genomic sequences when activating gene expression. To clarify the mechanism underlying the altered expression of myogenic regulatory genes in HP1α depleted myocytes, we examined if HP1α directly associates with the genomic sequences of *Lbx1*. Lbx1 plays a critical in hypaxial muscle development [21], [22]. It has been reported that *Lbx1* functions upstream of MyoD [23] and is thus the earliest myogenic gene whose expression is altered by HP1α-deficiency. We performed ChIP assays using primers that span the entire *Lbx1* genomic sequence including putative promoter, exons and intronic regions (Fig. 5A). Primers within the promoter of the *Col11a2* gene, a known HP1α target, were used as a positive control [24]. As shown in Figure 5B, endogenous HP1α binds preferably to exon 2 of *Lbx1* in myoblasts while the binding of HP1α to the other genomic sequences of *Lbx1* was undetectable. HP1 has been shown to stimulate gene expression in Drosophila and these HP1-induced target genes were marked by the presence of dimethylated histone 3 lysine 4 (H3K4me2), which is found, along with histone acetylation (H3K9Ac), on active chromatin [25]. To determine the effects of HP1α depletion on histone modifications present on *Lbx1* exon 2, we examined the status of H3K9Ac and H3K4me2 in C2C12 myoblasts transfected with NSsiRNA and HP1αsiRNA. Surprisingly, there were no significant changes in levels of H3K4me2 or H3K9Ac, however, levels of H3K9me3, typically associated with gene silencing were increased by 59% in HP1α depleted myoblasts as compared to control cells (Fig. 5C and Fig. S4A *P*<0.01). This effect was specific to exon 2 as H3K9me3 in exon 1 or the intronic region was unchanged (Fig. 5D). These data suggest that HP1α paradoxically might be mediating demethylation of H3K9me3 within exon 2 of *Lbx1* in myoblasts.

To investigate if the effect of HP1α on H3K9me3 is specific to the *Lbx1* gene or possibly a more general effect, we compared the levels of total H3K9me3 in C2C12 myoblasts transfected with NSsiRNA and HP1αsiRNA. HP1α deficient cells have higher levels of H3K9me3 (Fig. 5E), suggesting that HP1α regulates global H3K9me3 in myoblasts. Consistent with the downgregulation of myogenin expression after knockdown of HP1α, H3K9me3 levels at the myogenin promoter were higher in HP1αsiRNA transfected myoblasts compared to NSsiRNA transfected cells (Fig. 5F).

### HP1α Promotes Myogenic Gene Expression via Interacting with the Histone Demethylase JHDM3A

**Figure 6:**
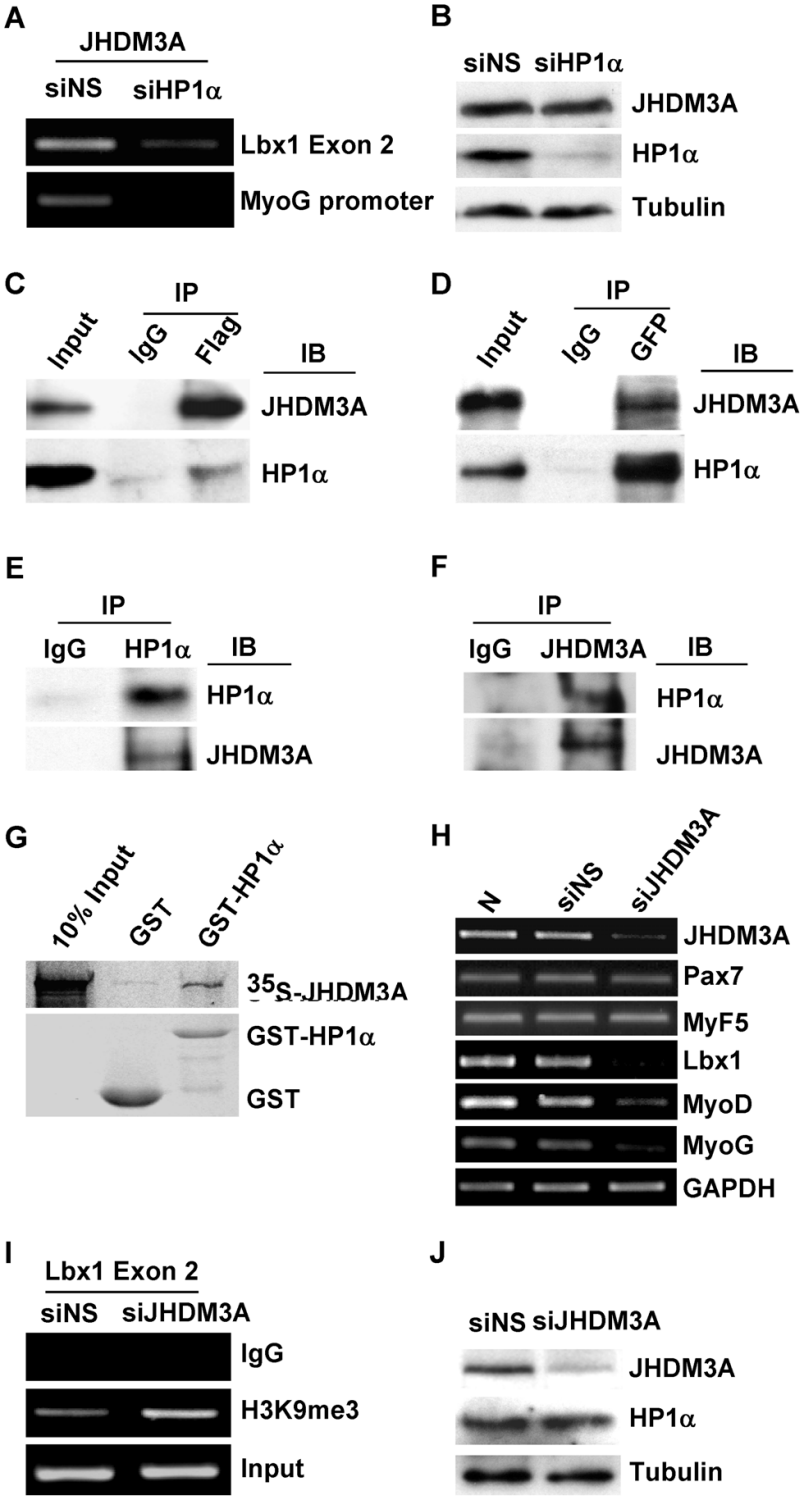
HP1α recruits JHDM3A to *Lbx1* gene and knockdown of JHDM3A in myoblasts impairs Lbx1, MyoD and myogenin expression. *A.* C2C12 myoblasts were transfected with NSsiRNA or HP1αsiRNA, 48 hours after transfection ChIP assay was performed with anti-JHDM3A antibody. *Lbx1* exon 2 and myogenin gene promoter were amplified. *B.* C2C12 myoblasts were transfected with the indicated siRNA, 48 hours after transfection total cell lysates were subjected to Western blotting with the specified antibodies. *C, D* Immunoprecipitation (IP) was performed using nuclear extracts prepared from NIH3T3 cells over-expressing Flag-JHDM3A and GFP-HP1α with either anti-Flag (C) or anti-GFP antibodies (D). Immunoprecipitated complexes were blotted (IB) with indicated antibodies. *E, F.* Endogenous HP1 and JHDM3A interaction was examined using C2C12 myoblasts nuclear extract. Nuclear extracts were immunoprecipitated with anti-HP1α (E) or anti-JHDM3A (F) antibody, and western blotted for HP1α and JHDM3A. *G*, *In vitro* translated ^35^S-JHDM3A protein was incubated with GST or GST-HP1α immobilized on the glutathione beads. Bound proteins were separated on SDS-PAGE and visualized by autoradiography. *H.* C2C12 myoblasts cultured in GM were transfected with NSsiRNA or JHDM3AsiRNA. After 48 hours, total RNA was isolated and expression of the indicated genes determined by semiquantitative PCR. *I.* C2C12 myoblasts were transfected with NSsiRNA or JHDM3AsiRNA, 48 hours after transfection cross-linked chromatin was extracted and immunoprecipitated with anti-H3K9me3 antibody. *Lbx1* exon 2 genomic sequences were amplified. *J.* C2C12 myoblasts were transfected with the indicated siRNA, 48 hours after transfection total cell lysates were subjected to Western blotting with the specified antibodies.

It has been recently reported that the mammalian demethylase, JHDM3A, is capable of removing the me3 group from modified H3K9 and H3K36 [26], [27], [28]. To determine if an interaction between HP1α and JHDM3A might account for HP1α’s paradoxical effect on transcription and methylation of myogenic genes, we examined whether JHDM3A could also associate with these genes in myoblasts. ChIP assays performed on myoblasts transfected with either NSsiRNA or HP1αsiRNA demonstrated that endogenous JHDM3A is also present at the *Lbx1* exon 2 and myogenin promoter, and that this association is impaired in HP1α deficient cells (Fig. 6A and Fig. S4B). The impairment of JHDM3A binding was not due to decreased protein levels in HP1α depleted cells (Fig. 6B). To ascertain if JHDM3A associates with HP1α, we overexpressed Flag-JHDM3A and GFP-HP1α in NIH3T3 cells and immunoprecipitated the resultant lysates with either anti-Flag (Fig. 6C) or anti-GFP antibodies (Fig. 6D). Western blotting of the precipitated complexes with anti-HP1α or anti-JHDM3A antibodies revealed that JHDM3A associates with HP1α. Co-immunoprecipitation analyses on lysates prepared from unmodified C2C12 cells confirmed that endogenous HP1α and JHDM3A can also associate *in vivo* (Fig. 6E and F). We further confirmed HP1α-JHDM3A interaction using *in vitro* binding assays. *In vitro* translated ^35^S-methionine-labeled JHDM3A interacted with a recombinant GST-HP1α fusion protein (Fig. 6G). If HP1α recruitment of JHDM3A to *Lbx1* exon 2 were required for efficient gene expression we would expect that depletion of JHDM3A in myoblasts would recapitulate the effects of HP1α deficiency, at least on *Lbx1, MyoD* and myogenin transcription. C2C12 cells were transfected with JHDM3AsiRNA and knockdown efficiency was confirmed by examining JHDM3A mRNA levels by RT-PCR (Fig. 6H). Consistent with the effect of depleting HP1α on myogenic genes expression, JHDM3A depletion led to a marked reduction in *Lbx1, MyoD* and myogenin transcript levels in myoblasts (Fig. 6H), which subsequently normalized with differentiation (Fig. S5A). We did not detect significant change of mRNA levels of Pax7 and Myf5 in JHDM3A knockdown cells (Fig. 6H). We further examined the H3K9me3 status on exon 2 of *Lbx1* and detected higher levels of H3K9me3 in JHDM3AsiRNA transfected myoblasts compared to NSsiRNA transfected cells (Fig. 6I). Knockdown of JHDM3A did not affect HP1α expression (Fig. 6J). Taken together, these results support a model whereby HP1α facilitates early myogenic gene expression in myoblasts through recruitment of JHDM3A to target genes.

## Discussion

In this study, we demonstrated that among the three isoforms of HP1, HP1α was specifically required for myogenic gene expression in myoblasts. Superficially, this data appears at odds with recent reports that demonstrated HP1 proteins negatively regulate the transcriptional activity of MyoD and MEF2C [12], [16]; however, HP1’s effect on endogenous myogenic genes was not examined in these studies. Instead, consistent with our results, it was reported that down-regulation of HP1α impaired differentiation. The molecular basis for this paradox was postulated that this effect related to a loss of HP1α-dependent suppression on expression of proliferation-associated genes [16]. We did not detect significant upregulation of E2F-dependent target genes after depletion of HP1α in C2C12 myoblasts, thus failure to downregulate proliferation associated genes could not account for this finding. In contrast, depletion of HP1α blocked expression of myogenic genes even before differentiation induction (Fig. 2C). The defect in skeletal muscle differentiation in HP1α-depleted myoblasts was accompanied by reduced Lbx1, MyoD, MEF2C and myogenin expression without significantly effecting Pax7 or Myf5. Reduction in expression of these key regulators of myogenesis is likely sufficient to account for the subsequent defect in myogenic differentiation we observed. This dependence of skeletal muscle differentiation was specific to myoblasts since no defect was seen when HP1α was knockdown in myotubes. This data is reminiscent of recent studies exploring Rb’s role in skeletal muscle differentiation, where Rb is critical for progression through myogenic differentiation [29], [30], [31] but is dispensable for the maintenance of the terminally differentiated state [32], [33]. Although our study demonstrates that HP1α is dispensable for maintenance of terminal differentiation, whether this implies HP1α has no role in myotubes or whether HP1β and γ, which are expressed normally in HP1α deficient myotubes, compensate for HP1α’s function will need to be determined. Although overexpression of a siRNA resistant HP1α was able to rescue the defect in myogenic gene expression in transfected cells there was a persistent defect in myotube formation (Fig. 2). Although we achieved very high transfection efficiency (>90%) for siRNA in C2C12 cells, the transfection efficiency of pLPC-EHGF-rHP1 alpha in C2C12 is low but consistent with published transient transfection rates in these cells [34]. Thus, we can not determine if the defect in myotube formation is related to the expected low rate of rescue due to low transient transfection efficiency, high levels of HP1α expression by transfected construct versus a true physiologic difference.

Although HP1’s ability to silence gene transcription is well documented, few examples exist of its ability to activate gene expression in mammalian cells and little is known regarding the possible mechanisms. However, it was known that when activating gene expression HP1 can associate anywhere within genomic sequences [9]. It has been recently demonstrated that Pax7 activates Myf5 expression by recruitment of a histone methytransferase complex to Myf5 coding regions [35]. However HP1α’s function on transcription activation appeared independent of histone acetyltransferases and methyltransferase since there was no discernable change in the level of H3K9Ac and H3K4me2 after knockdown of HP1α expression. Instead, we found total H3K9me3 levels increased after knockdown of HP1α either in promoter or transcribed regions generally is associated with gene silencing [36]. Depleting HP1α decreased Lbx1 expression in C2C12 myoblasts correlating with increased H3K9me3 on *Lbx1* exon 2. This appears to be a direct effect since HP1α binds directly to this same region of *Lbx1*. Interestingly, despite previous reports that HP1α interacted with MEF2C to inhibit myogenin expression, depleting HP1α resulted in decreased endogenous myogenin expression, which was associated with a higher level of H3K9me3 at the myogenin promoter. These results suggest that HP1α might be promoting demethylation of H3K9me3 at myogenic promoters.

Recent studies have identified a family of JmjC domain-containing proteins that possess histone demethylation activity and facilitate transcription activation [26], [27], [28]. We were able to demonstrate an association of JHDM3A with genomic sequence of *Lbx1* and *myogenin* and this interaction was HP1α-dependent. JHDM3A, also known as JMJD2A, is a JmjC domain-containing protein that possesses histone demethylation activity, which specifically demethylates tri- and di- methylation of H3K9 [26], [27]. We demonstrated that JHDM3A interacts with HP1α and the effects of JHDM3A knockdown on myogenic gene transcription were similar to HP1α deficiency. These results suggest that HP1α recruits JHDM3A to transcriptional regions of *Lbx1*, demethylating H3K9me3, and facilitating transcription to promote myogenic differentiation. However, knockdown of JHDM3A delayed myogenic gene expression but did not block C2C12 differentiation completely (Fig. S5 and data not shown). Several possible explanations exist for this observation. JHDM3A may be critical early for myogenic gene transcription but other mechanisms predominate later in skeletal differentiation. Alternatively, there are other demethylase(s) that can compensate for the lack of JHDM3A function in differentiating myocytes. JHDM3A along with JMJD2B, C and D belong to the JmjC domain-family of histone demethylase. JHDM3A, JMJD2C and JMJD2D are all capable of demethylating tri-methylated H3K9 [17]. Although we did not detect JMJD2D in C2C12 cells, JMJD2C is expressed in C2C12 cells and knockdown of JHDM3A with siRNA did not affect JMJD2C expression.

Methylation of H3K9 has been strongly implicated in HP1 recruitment and formation of heterochromatin [28]. Thus, the interaction of HP1 with histone deacetylases and methytransferases has been well studied [12], [37]. However, there is little data related to the interaction of HP1 with demethylases in mammalian cells. It has been reported that Swi6, a homolog of HP1 in yeast, recruits Epe1, a JmjC domain protein, to heterochromatin loci to facilitate transcription [38]. Recently Lin et al [39] reported that HP1α specifically interact with the Drosophila KDM4A demethylase and stimulates histone H3 lysine 36 demethylation. Our study is the first to suggest that similar interaction between HP1α and the demethylase JHDM3A occur in mammalian cells, suggesting a new paradigm for the regulation of tissue-specific gene expression.

Our study proposes a novel function for HP1α in maintenance of myogenic gene expression in myoblasts by inhibiting H3K9me3 via interacting with JHDM3A, which is consistent with previous findings that HP1 can activate gene expression in Drosophila [9], [40]. HP1α has also been reported to inhibit MEF2-dependent transcription by interacting with MITR and HDAC9 to form a potent transcriptional repressor complex in undifferentiated myoblasts [12]. Thus the roles of HP1 family members in differentiation are likely complex. HP1 may play multiple, developmentally dependent functions in differentiation, and it’s positive versus negative transcriptional effects might be determined by interacting partners. The basis for specificity in recruitment of these partners is unknown at this time; however, all three HP1 isoforms can be heavily modified and these posttranslational modifications might modulate HP1 interactions with silencing and antisilencing proteins [41]. Additionally, both HP1 and JHDM3A proteins levels are regulated during myogenic differentiation (Fig. 1A and Fig. S5B). Clearly epigenetic regulation and post-translational modifications of histone play an important role in skeletal muscle differentiation and it will be important to better understand the complex regulation of these factors.

## Materials and Methods

### Cell Culture and Plasmids

Mouse C2C12 cells (ATCC) were propagated as myoblasts in DMEM containing 10% fetal bovine serum (Growth Medium, GM) and 1% penicillin G and streptomycin sulfate (Invitrogen) at 37°C in a humidified 5% CO2 incubator and induced to differentiate using DMEM containing 2% horse serum (Differentiation medium, DM). NIH3T3 cells were grown in DMEM containing 10% NCS and 1% penicillin G and streptomycin sulfate. pLPC-EHGF-HP1α was kindly provided by Dr. Scott Lowe from Cold Spring Laboratories. pCDNA3-Flag-JHDM3A was kindly provided by Dr. Yi Zhang from Howard Hughes Medical Institute. The plasmid containing the siRNA resistant version of HP1α was generated via a site-directed mutagenesis kit (Stratagene) using pLPC-EHGF-HP1α as a template. To evade RNA silencing, four nucleotides within the HP1α siRNA target sequence were mutated. This mutation (TTTTCT to TTCAGC) does not change the encoded amino acids.

### Immunostaining

C2C12 cells were fixed with 4% paraformaldehyde and immunostained with antibodies to HP1α (15.19s2, Upstate Biotech, Inc.), HP1β (MAB3448, Chemicon), HP1γ (MAB3450, Chemicon), Sarcomeric myosin heavy chain (Developmental Hybridoma Studies Bank), GFP (Santa Cruz) and Troponin C (Santa Cruz). Cells were counter-stained with DAPI and Phallotoxins (Molecular Probes).

### RNA and Protein Analysis

Total RNA was isolated from C2C12 cells using Tri Reagent (Sigma). Semi-quantitative RT-PCR was performed as described [42]. Primers sequences are available upon request. Western blotting and immunoprecipitation were performed according to standard procedures. Nuclear extracts were prepared as described [43]. Antibodies to HP1α (Upstate Biotech, Inc.), HP1β (MAB3448, Chemicon), HP1γ (MAB3450, Chemicon), Myogenin (F5D, BD Biosciences), α-skeletal actinin (Sigma), phosphor-Histone (Ser10) (Upstate Biotech, Inc.), Tubulin (Sigma), GFP (Abcam), Flag (Sigma), CDC2, p21, JHDM3A (Bethyl laboratories) and Laminin B (Santa Cruz Biotech) were purchased commercially. In vitro binding assays were performed according to standard procedures as described [44]. pGEX-KG-HP1α was kindly provided by Dr. Eric N. Olson.

### RNA Interference

C2C12 cells were transfected with 100 nM of HP1αsiRNA (ID# 60497 or ID# 60593 Ambion, ID# 60497 was used unless otherwise noted), HP1βsiRNA (ID# 160032, Ambion), HP1γsiRNA (ID# 160038, Ambion), JHDM3AsiRNA (Cat# SI00241003 Qiagen) or nonspecific siRNA (Cat# 1027310, Qiagen) was transfected into cells using Lipofectamin 2000 (Qiagen).

### Chromatin Immunoprecipitation (ChIP) and Real-time Quantitative PCR

PCR-ChIP analysis was performed as described [44]. Chromatin was incubated at 4°C overnight with antibodies specific to HP1α (Upstate Biotech, Inc.), H3K9me3 (Upstate Biotech, Inc.), H3K9Ac (Upstate Biotech, Inc.), H3K4me2 (Upstate Biotech, Inc.) and JHDM3A (Bethyl Lab. Inc), normal mouse or rabbit IgG was used as control. PCR was performed using Platinum Taq polymerase (Invitrogen). The following pairs of primers were used for PCR: Lbx1 putative promoter: P-S (AGGAGAAAGAAGGGAGAGCGAAGA), P-AS (ATTTAAAGGTGTCAGGTGGCTCCG) Lbx1 Exon 1: E1-S (5′- AAACGAGGCCGAGATGACTTCCAA-3′), E1-AS (5′- AGAGAGGCGAGGTCTGCGA-3′); Lbx1 intron: I-S (5′- TCCGCTTGGACTCTCCTAGACTTA-3′), I-AS (5′- CACAGAGTGCAAGGAAACCACACA-3′); Lbx1 Exon 2: E2-S (5′- TCGTCGCGAGCATACACTGTCATT-3′), E2-AS (5′- TGATGACCTGTGCATTGGTGAGG -3′); for Col11a2: C1 (5′-GGATGCTGCCACGGCCTGAGG-3′), C2 (5′-GGGTCTGCCAGGAGCCTGTGG-3′) [24], for myogenin promoter: [+](5′-GAATCACATGTAATCCACTGGA-3′; [−](5′-ACGCCAACTGCTGGGTGCCA-3′) [45]. Immunoprecipitated DNA from three independent ChIP analyses with anti-H3K9me3 antibody was subjected to real-time quantitative PCR in triplicate. Real-time quantitative PCR was conducted using the ABI PRISM 7700 Sequence Detection System; Taqman (Applied Biosystems, Foster City, CA). PCR amplicons were detected by fluorescent detection of SYBR Green (QuantiTect SYBR Green PCR Kit, Qiagen). Primers sequence for *Lbx1* Exon 2 is listed above.

### BrdU Incorporation Assay

C2C12 cells were incubated in DM for 72 hours and transfected with 100 nM oligonucleotide siRNAs. 24 hours after transfection, cells were cultured with DMEM containing 20% FBS in the presence of BrdU labeling reagent. Then cells were subjected to BrdU (Roche) and sarcomeric myosin heavy chain immunofluorescent staining, nuclei were counter-stained with DAPI according to the manufacturer’s instructions.

### EDU Cell Proliferation Assay

EDU (5-ethynyl-2′-deoxyuridine) assay were performed using Click-iT® EdU HCS Assays (Invitrogen). C2C12 cells were seeded at a density of 4×10^4^ cells/well on 24-well plate containing cover slips and cultured with 10% FBS DMEM overnight. And then transfected with 100 nM oligonucleotide siRNAs. 24 hours after transfection, cells are incubatedd with 10 µM EDU for 4 hr. The cells which incorporated EDU were stained following the manufacture’s instruction.

## Supporting Information

Figure S1 **Confocal fluorescence microscopy was performed on C2C12 myoblasts and myotubes after immunostaining for the indicated HP1 protein (green) and DAPI (converted to red).**
(PDF)

Figure S2 **C2C12 myoblasts were treated with EDU after indicated siRNA transfection.** The cells were then fixed and stained for EDU incoperation and HP1a expression. Nuclei were visualized by DAPI staining.
(PDF)

Figure S3 **C2C12 myoblasts were co-transfected with indicated siRNA and GFP-expressing plasmid.** GFP-expressing cells were isolated by FACS. Staining with 7-AAD was performed to exclude dead cells.
(PDF)

Figure S4 **C2C12 cells were transfected with nonspecific siRNA (siNA) or HP1αsiRNA (siHP1α).** 48 hours after transfection Chip assay was performed with anti-H3K9me3 (A) or anti-JHDM3A antibody (B). H3K9me3 levels (A) and presence of JHDM3A (B) at Lbx1 exon 2 were quantified by Real time PCR. *P<0.01 for siNS transfected cells versus siHP1α transfected cells.
(PDF)

Figure S5 A. C2C12 cells were transfected with nonspecific siRN or JHDM3AsiRNA. Total RNA was isolated at indicated time points after transfection. Semiquantitative PCR analysis of gene expression was performed. B. Western blotting was performed with indicated antibodies on whole proteins extracted from C2C12 myoblasts (MB) and myotubes (MT).
(PDF)
