# Supplementary material for: Epigenetic Regulation of Myogenic Gene Expression by Heterochromatin Protein 1 Alpha
Source: PLoS One. 2013 Mar 11;8(3):e58319. doi: 10.1371/journal.pone.0058319 (PMC3594309; doi:10.1371/journal.pone.0058319)
Supplement: Figure S1 — Confocal fluorescence microscopy was performed on C2C12 myoblasts and myotubes after immunostaining for the indicated HP1 protein (green) and DAPI (converted to red). (PDF) [file pone.0058319.s001.pdf]

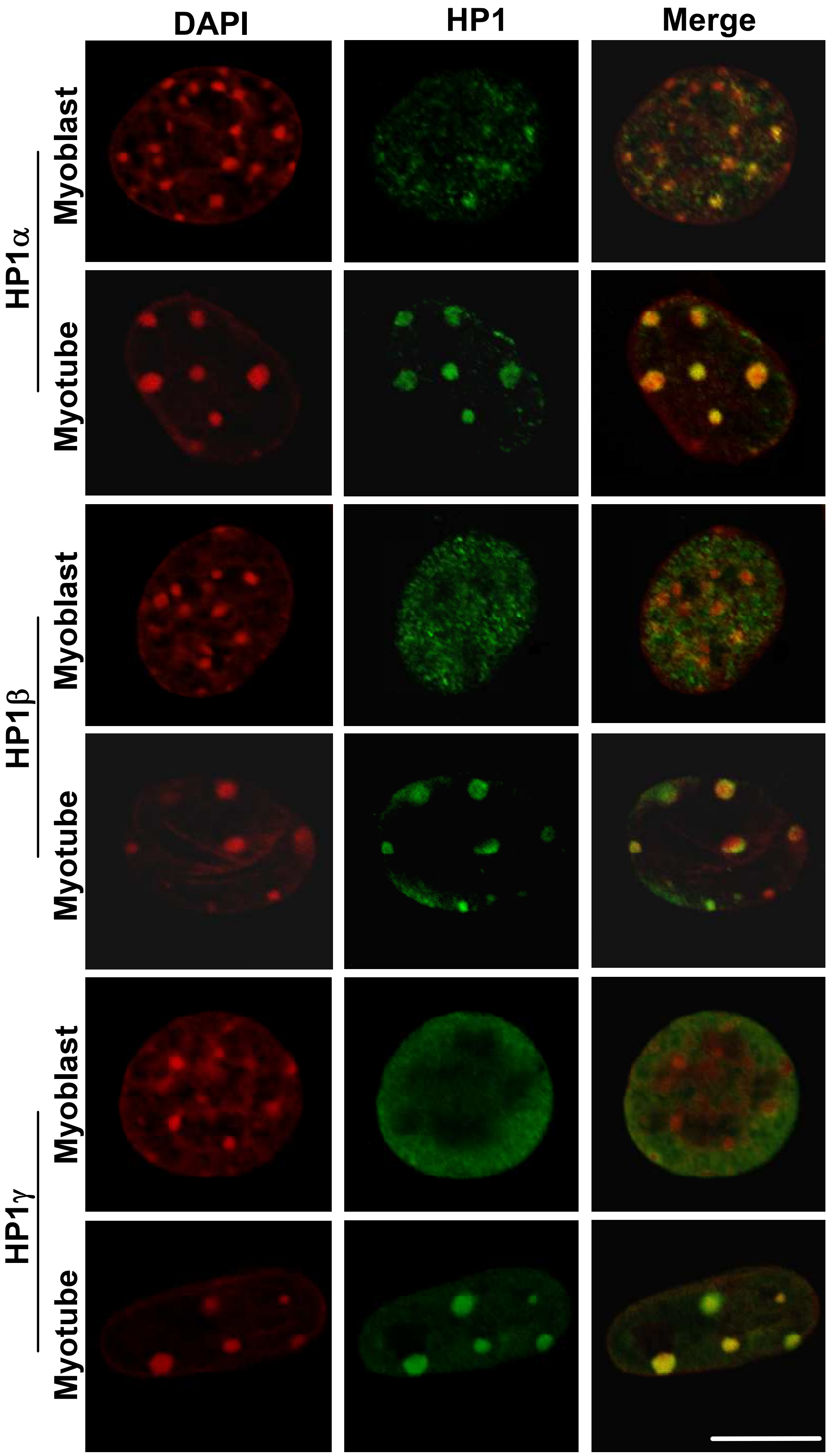

Fig. S1. Confocal fluorescence microscopy was performed on C2C12 myoblasts and myotubes after immunostaining for the indicated HP1 protein (green) and DAPI (converted to red). Scale bar equals 10μm.

Fig. S1 Sdek et al
