# Supplementary material for: Epigenetic Regulation of Myogenic Gene Expression by Heterochromatin Protein 1 Alpha
Source: PLoS One. 2013 Mar 11;8(3):e58319. doi: 10.1371/journal.pone.0058319 (PMC3594309; doi:10.1371/journal.pone.0058319)
Supplement: Figure S2 — C2C12 myoblasts were treated with EDU after indicated siRNA transfection. The cells were then fixed and stained for EDU incoperation and HP1a expression. Nuclei were visualized by DAPI staining. (PDF) [file pone.0058319.s002.pdf]

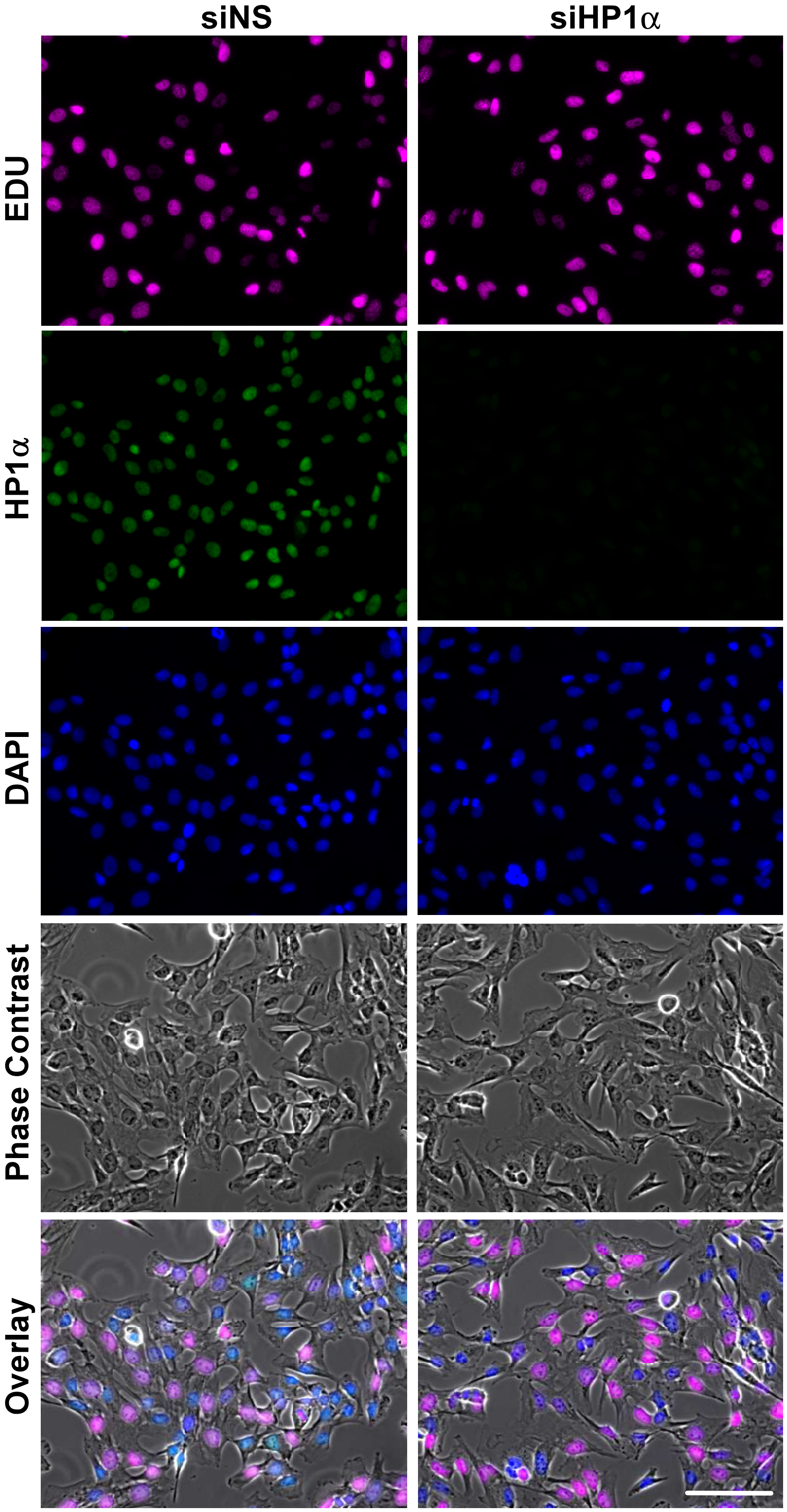

Fig. S2 C2C12 myoblasts were treated with EDU after indicated siRNA transfection. The cells were then fixed and stained for EDU incoperation and HP1α expression. Nuclei were visualized by DAPI staining. Scale bar: 100μm

Fig. S2 Sdek et al
