# Supplementary material for: Epigenetic Regulation of Myogenic Gene Expression by Heterochromatin Protein 1 Alpha
Source: PLoS One. 2013 Mar 11;8(3):e58319. doi: 10.1371/journal.pone.0058319 (PMC3594309; doi:10.1371/journal.pone.0058319)
Supplement: Figure S3 — C2C12 myoblasts were co-transfected with indicated siRNA and GFP-expressing plasmid. GFP-expressing cells were isolated by FACS. Staining with 7-AAD was performed to exclude dead cells. (PDF) [file pone.0058319.s003.pdf]

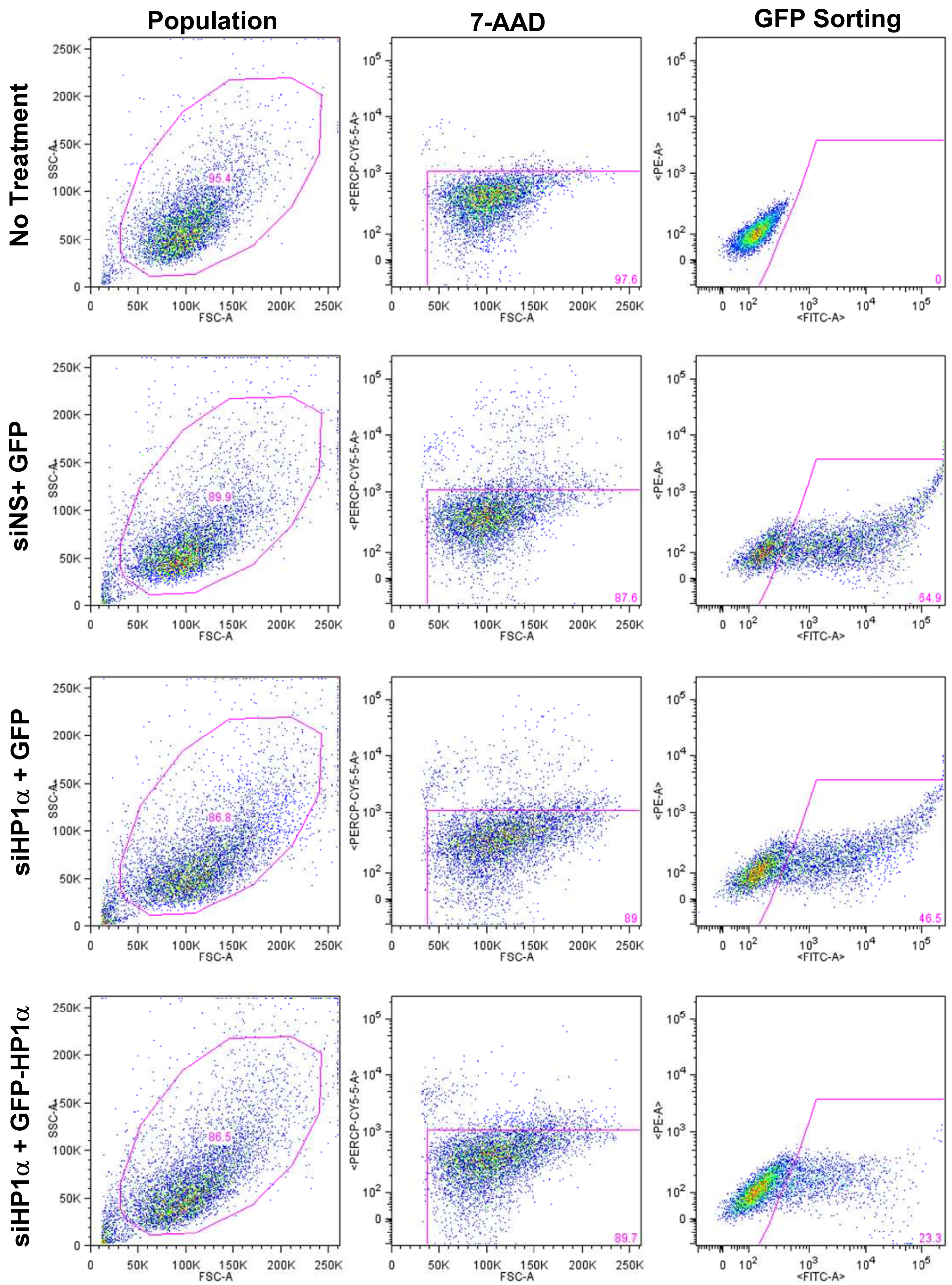

Fig. S3 C2C12 myoblasts were co-transfected with indicated siRNA and GFP-expressing plasmid. GFP-expressing cells were isolated by FACS. Staining with 7-AAD was performed to exclude dead cells.

Fig. S3 Sdek et al
