# Supplementary material for: Epigenetic Regulation of Myogenic Gene Expression by Heterochromatin Protein 1 Alpha
Source: PLoS One. 2013 Mar 11;8(3):e58319. doi: 10.1371/journal.pone.0058319 (PMC3594309; doi:10.1371/journal.pone.0058319)
Supplement: Figure S4 — C2C12 cells were transfected with nonspecific siRNA (siNA) or HP1αsiRNA (siHP1α). 48 hours after transfection Chip assay was performed with anti-H3K9me3 (A) or anti-JHDM3A antibody (B). H3K9me3 levels (A) and presence of JHDM3A (B) at Lbx1 exon 2 were quantified by Real time PCR. *P<0.01 for siNS transfected cells versus siHP1α transfected cells. (PDF) [file pone.0058319.s004.pdf]

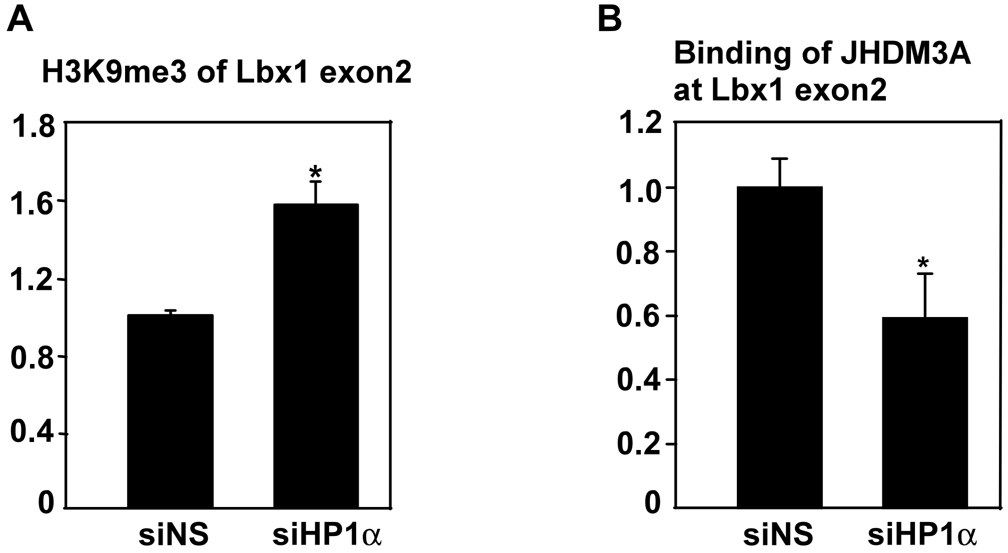

Fig. S4 C2C12 cells were transfected with nonspecific siRNA (siNA) or HP1αsiRNA (siHP1α). 48 hours after transfection Chip assay was performed with anti-H3K9me3 (A) or anti-JHDM3A antibody (B). H3K9me3 levels (A) and presence of JHDM3A (B) at Lbx1 exon 2 were quantified by Real time PCR . *P<0.01 for siNS transfected cells versus siHP1α transfected cells.

Fig. S4 Sdek et al
