# Supplementary material for: Epigenetic Regulation of Myogenic Gene Expression by Heterochromatin Protein 1 Alpha
Source: PLoS One. 2013 Mar 11;8(3):e58319. doi: 10.1371/journal.pone.0058319 (PMC3594309; doi:10.1371/journal.pone.0058319)
Supplement: Figure S5 — A. C2C12 cells were transfected with nonspecific siRN or JHDM3AsiRNA. Total RNA was isolated at indicated time points after transfection. Semiquantitative PCR analysis of gene expression was performed. B. Western blotting was performed with indicated antibodies on whole proteins extracted from C2C12 myoblasts (MB) and myotubes (MT). (PDF) [file pone.0058319.s005.pdf]

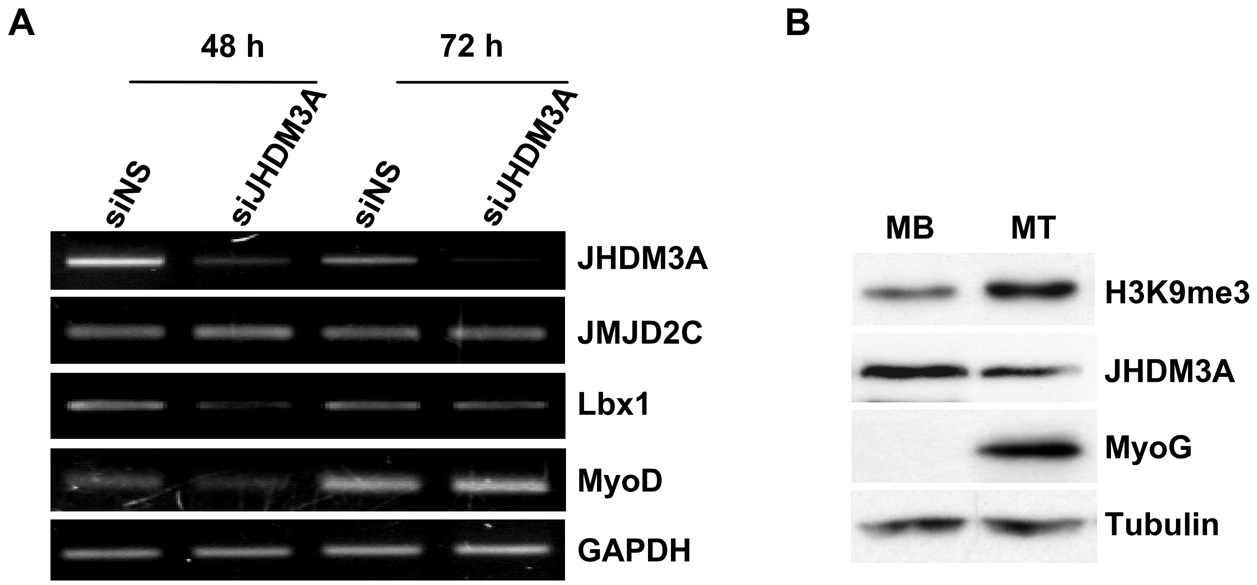

Fig. S5 A. C2C12 cells were transfected with nonspecific siRNA or JHDM3AsiRNA. Total RNA was isolated at indicated time points after transfection. Semiquantitative PCR analysis of gene expression was performed. B. Western blotting was performed with indicated antibodies on whole proteins extracted from C2C12 myoblasts (MB) and myotubes (MT).

Fig. S5 Sdek et al
